# Using mixed methods to establish tobacco treatment acceptability from the perspective of clients and clinicians of antenatal substance use services

**DOI:** 10.1186/s13722-022-00337-y

**Published:** 2022-10-04

**Authors:** Melissa A. Jackson, Penny Buykx, Amanda L. Brown, Amanda L. Baker, Adrian J. Dunlop, Gillian S. Gould

**Affiliations:** 1 Hunter New England Local Health District Drug and Alcohol Clinical Services, Level 3, 670 Hunter Street, Newcastle, NSW 2290 Australia; 2 School of Humanities, Creative Industries and Social Sciences, University of Newcastle, Callaghan, NSW Australia; 3 School of Medicine and Public Health, University of Newcastle, Callaghan, NSW Australia; 4 Faculty of Health, Southern Cross University, Lismore, NSW Australia

**Keywords:** Tobacco treatment, Substance use disorders, Tobacco intervention, Smoking cessation, Pregnancy, Antenatal care, Harm reduction, Mixed methods, Health services, Public health

## Abstract

**Background:**

Up to 95% of pregnant women with alcohol and other drug (AOD) problems also smoke tobacco. Challenging psychosocial circumstances and a lack of targeted tobacco interventions contribute to low rates of prenatal abstinence and more effective treatment strategies are required. This study explores smoking in pregnant clients of AOD treatment services from a consumer and healthcare provider perspective to examine characteristics of behaviour change and the acceptability of evidence-based tobacco treatment strategies. Outcomes will support the design and implementation of a comprehensive tobacco intervention.

**Methods:**

A mixed methods triangulated design was used. Thirteen women who smoked and attended antenatal AOD services in New South Wales, Australia, were interviewed and 28 clinicians from the same services were surveyed. Domains including experiences of tobacco smoking in pregnancy, motivators and barriers to cessation and evidence-based strategies to assist cessation during pregnancy were explored. Interviews were analysed using Iterative Categorization, with interpretation guided by Qualitative Description. Online surveys were analysed descriptively. A convergent-parallel mixed methods analysis was performed.

**Results:**

Women and clinicians agreed that improving baby’s health outcomes was the primary motivation to stop smoking. Negative experiences with nicotine replacement therapy (NRT), financial constraints and maternal contraindications restricted its uptake and effectiveness during pregnancy. Both groups agreed that other AOD use, stopping multiple substances concurrently, difficulty coping with stress and the influence of partners who smoke had the biggest impacts on cessation efforts. Clinicians favoured harm-reduction rather than abstinence-based tobacco interventions and women appeared satisfied with reduction efforts. Both views may influence the attainment of prenatal abstinence-based goals. Although previous evidence suggested the contrary, clinicians were willing to encourage simultaneous cessation of tobacco and other substances. Non-judgmental treatment approaches that provide extra support, education and motivation were important for women. Women and clinicians supported use of NRT despite concerns. Financial incentives, counselling, partner support and offering tobacco treatment with antenatal AOD care were considered acceptable treatment options.

**Conclusions:**

NRT, incentives, counselling and partner support could be utilized in a tobacco intervention for pregnant women with substance use concerns. Non-judgmental education, motivation, and provision of NRT including instruction for correct use are important considerations.

**Supplementary Information:**

The online version contains supplementary material available at 10.1186/s13722-022-00337-y.

## Background

Tobacco smoking in pregnancy is well established as a major risk factor for adverse maternal and fetal health outcomes. In pregnancy, smoking has been strongly associated with intrauterine growth restriction, ectopic pregnancy, placental abruption, placenta previa, pre-term birth, miscarriage and stillbirth [1, 2]. For offspring, the consequences of prenatal cigarette smoke exposure are extensive, with infants more likely to experience low birthweight, sudden unexpected death in infancy, chronic respiratory disorders, cardiovascular disease, obesity, attachment difficulties and learning and behavioural difficulties [2–4]. Smoking also increases the likelihood of exposed infants developing future tobacco and other substance use disorders [5, 6].

To protect themselves and their offspring, many women stop smoking before conceiving, or within the first two trimesters of pregnancy [7]. Adverse psychosocial circumstances contribute to some women having difficulties achieving abstinence, despite having a maternal desire to do so. A number of women will be from disadvantaged populations, including those with alcohol or other drug (AOD) concerns [8]. Smoking rates in this group are particularly elevated during pregnancy, with prevalence estimates of between 71 and 95% [9, 10]. Few tobacco interventions target this group [11] and contribute to low rates of abstinence. Moreover, other significant barriers to quitting have been identified, including comorbid mental illness and the use of tobacco smoking as a coping mechanism to deal with stress and other symptoms of mental illness [12]. Additionally, norms for smoking in close familial and social networks can negatively influence attempts to address smoking during pregnancy [13, 14]. Prohibitive systemic challenges include the prioritization of other substance use treatment during pregnancy [15] and the inability of antenatal services to provide the support required to address cessation in this group [13].

A range of tobacco treatments have been trialled with varying effectiveness in pregnant women, including pharmacotherapies (nicotine replacement therapy (NRT), bupropion and varenicline) [16, 17] and behavioural therapies (counselling, financial incentives, health education, feedback, social support and exercise) [18]. However, evidence of their efficacy in maternal AOD populations is scarce. The inadequate treatment of tobacco smoking for pregnant women with other substance use problems has been highlighted in a recent review [11]. A lack of sustained abstinence in participants of the seven included behavioural interventions was noted. Only one randomized controlled trial, utilizing financial incentives for verified smoking abstinence was able to demonstrate abstinence in their treatment group compared to controls at treatment-end, however this was not sustained post-treatment [19]. The review suggested that long-term tobacco treatments utilizing multiple evidence-based approaches, including financial incentives, may be helpful in this population.

With the paucity of targeted interventions, tobacco treatments for general AOD populations may provide direction. A 2016 Cochrane review of tobacco use interventions for people in treatment or post-treatment for substance use disorders [20], concluded that tobacco abstinence was increased by the provision of pharmacotherapy that included NRT, varenicline and bupropion (RR 1.88, 95%CI 1.35–2.57, 11 studies, 1808 participants, low-quality evidence) or combined counselling and pharmacotherapy (RR 1.74, 95%CI 1.39–2.18, 12 studies, 2229 participants, low-quality evidence) at the longest follow-up timepoints. It was also noted that tobacco treatment did not adversely impact AOD abstinence rates.

The outcomes of this study will be used to support the implementation and evaluation of a comprehensive tobacco intervention tailored to pregnant women with concurrent AOD and tobacco use. Underpinned by existing literature, several tobacco interventions with the potential to create sustained tobacco-related behaviour change in this high-risk population have been selected. The acceptability of financial incentives, NRT, telephone-based counselling and provision of support to smoking partners as individually delivered tobacco treatments will be pragmatically assessed from the perspective of clients and clinicians of substance use in pregnancy services. To further assist intervention development and contribute to the current literature, the motivators and barriers to smoking cessation and the experience of smoking during pregnancy will be explored. As far as we are aware, this is the first comprehensive investigation of smoking and influences on tobacco smoking behaviour change with consumer and healthcare provider input in Australian AOD antenatal settings.

## Methods

### Design and settings

The study used a mixed method triangulated design [21] to establish a meaningful picture of smoking, behaviour change and tobacco treatment in public AOD antenatal settings, including what a tailored smoking intervention might look like and how it would be received by both service providers and clients. In-depth interviews were conducted with women attending antenatal services dedicated to substance use in pregnancy located in tertiary referral hospitals, covering one metropolitan and one regional Local Health Districts in New South Wales (NSW), Australia. Online surveys were sent to clinicians working in the same clinical area from eight NSW Local Health Districts covering metropolitan, regional and rural healthcare settings. Both settings and participants were purposively selected to ensure diversity and that enough information-rich sources could be collected in an area of health that is relatively small and specialized.

Qualitative interviews provide rich contextual data from a consumer’s viewpoint, while the quantitative survey of clinicians provides verification and gives a systemic perspective. The online survey also allows greater reach given the small numbers of antenatal clinicians with AOD specialization. A convergent-parallel approach was used where both sets of data were collected, analysed and interpreted separately but concurrently, then merged at the interpretation level according to theme [21]. All authors have substantial experience using qualitative and quantitative research methods and all were involved in the integration of results, which occurred prior to manuscript development. The Consolidated criteria for reporting qualitative research (COREQ) [50] and Mixed methods studies in health services research reporting guidelines (GRAMMS) [51] were followed to ensure clear reporting of study methods and results. (See Additional file 1 - COREQ checklist and Additional file 2 - GRAMMS checklist)

### Women’s Interviews

#### Qualitative approach

The qualitative portion of this study was guided by Qualitative Description, a pragmatic, low-inference method that is in turn guided by naturalistic enquiry [22]. Its widespread use in health research is particularly useful for clinical intervention and questionnaire development [23].

#### Interview design

Interviews were semi-structured, containing open-ended questions and prompts to elicit a range of feedback (see Additional file 3 - Women's interview schedule). They explored a predetermined set of themes that included: (i) experiences of smoking; (ii) smoking in pregnancy; (iii) smoking cessation; and (iv) strategies that might assist cessation during pregnancy.

#### Recruitment

Women attending participating antenatal clinics were approached by clinicians regarding interview participation after smoking status was ascertained. Given the narrow focus of the research topic and sample sizes employed in similar qualitative description studies, it was expected that between 10 and 20 participants would be required to generate rich data [24].

Informed consent was obtained prior to commencement. Women were assured verbally and in the written information provided that participation was voluntary, and their future treatment would not be impacted by not taking part. All received $A40 retail gift voucher for reimbursement of the time and expense of attending the interview.

#### Data collection

Interviews occurred in outpatient healthcare settings between January and December 2019, taking 30 to 60 min to complete. All were audio-recorded to aid transcription. Demographic and substance use questions relating to past and current tobacco and AOD use were also completed.

Interviews were conducted in person by female research team members: a PhD candidate with a background in behavioural science (MAJ) and an occupational therapist with experience conducting quantitative research (NW). Both had AOD clinical experience. They introduced themselves and informed participants that the research was being conducted to aid the development of an antenatal tobacco treatment for women with AOD concerns prior to the interview.

Researchers discussed the interview process and their expectations of participants before interviews commenced. Reflective notes were made post-interview with interview technique and content reviewed together. These helped improve rigor and data consistency by refining interview and probing skills, reducing assumptions and avoiding data over-interpretation.

#### Analysis

After thirteen interviews, researchers determined that little new information was being added to the codes already in use, data saturation had been achieved. The raw data was transcribed verbatim using professional transcription services and reviewed by MAJ before being entered into NVivo version 12 software for coding. Double coding was undertaken to aid interpretation and increase rigor. The initial five interviews were independently coded by MAJ and PB, with patterns and themes compared, documented and refined. Any discord was discussed until agreement was reached. The resulting coding structure was primarily derived deductively, based on the structured interview guide, and inductively as new topics emerged (see Additional file 4 - Women's interview codebook). The study’s deductive nature and small number of interviews allowed remaining interviews to be coded by MAJ, with ongoing review and discussion with PB. Coded data was analysed using Iterative Categorization, [25] a systematic and transparent data management technique developed in the field of addiction research.

### Clinicians survey

#### Survey design

The 40-item survey explored clinicians’ perceptions of maternal smoking among their clients (see Table 4 in Results). Developed by the authors, it collected demographic information and covered four domains: (i) clinicians’ confidence and beliefs regarding smoking behaviour change; (ii) motivations to stop smoking; (iii) barriers to cessation; and (iv) characteristics of effective interventions. A 5-point Likert scale with a neutral option was used for the first domain to assess level of agreement with statements. Others used 4-point scales assessing frequency or effectiveness items and offered an open-ended option for further comments. Provision for additional comments or suggestions was also included.

#### Recruitment

Eligible participants were identified by their work roles and defined as anyone who reviewed women with AOD concerns during pregnancy in a specialized antenatal clinic. A designated contact at each participating antenatal service forwarded survey links to appropriate clinicians.

#### Data collection

Data was collected anonymously between August and December 2019 and the survey took 20–30 min to complete. A link to the approved information sheet was provided and informed consent implied upon survey completion.

#### Analysis

Descriptive analyses were conducted using Microsoft Excel. Where response options provided a graduated level of agreement or disagreement, they were recategorised to positive or negative. ‘Strongly agree’ and ‘agree’ responses and ‘strongly disagree’ and ‘disagree’ responses were combined and ‘sometimes’ and ‘always’ responses and ‘never’ and ‘rarely’ responses were combined.

## Results

### Women’s interviews

Demographic and smoking data for the 13 participants are summarized in Tables 1 and 2 respectively. Pseudonyms, not actual names, have been used for participant characteristics and quotations.

**Table 1:** Women’s demographics (n = 13)

Pseudonym	Age	Education completed	Income source	Relation ship (Y/N)	Children (n)	Children in mothers care (n)	Gestation (weeks)	Other substance/s prescribed or used during pregnancy
#1 Nicole	37	Year 10	Gov’t support	Yes	0	0	18	Cannabis
#2 Kate	45	Year 12	Gov’t support	Yes	1	1	10	Buprenorphine/Heroin
#3 Miranda	38	TAFE certificate	Gov’t support	No	1	1	24	Cannabis
#4 Amelia	34	TAFE certificate	Gov’t support	Yes	3	2	22	Cannabis
#5 Briana	27	Up to Year 9	Gov’t support	No	3	1	21	Methadone
#6 Sarah	31	Up to Year 9	Gov’t support	No	1	0	27	Methamphetamine
#7 Peta	36	TAFE certificate	Gov’t support	No	2	2	37	Cannabis
#8 Rose	31	Year 12	Gov’t support	Yes	3	0	28	Heroin/ Methadone
#9 Sofie	33	Bachelors	Part-time work	Yes	0	0	34	Cannabis/ Alcohol
#10 Sam	26	Up to Year 9	Gov’t support	No	0	0	31	Cannabis
#11 Anna	34	Year 10	Gov’t support	No	1	0	29	Alcohol
#12 Grace	20	Year 12	Part-time work	Yes	0	0	17	Cannabis/ Alcohol
#13 Daniela	39	Year 10	Gov’t support	Yes	5 +	0	27	Methadone
Average	33						25

**Table 2:** Women’s smoking characteristics (n = 13)

Pseudonym	Age of smoking initiation	Cigarettes smoked per day	Time to first cigarette (mins)	Nicotine dependence level^a^	Current smoking vs pre-pregnancy	Smokers in house-hold (n)	Household smoking rules^b^	Partner smoking since pregnancy
#1 Nicole	15	11–20	6–30	Moderate	Reduced	2	Just outside	Reduced
#2 Kate	10	≤ 10	6–30	Low	Reduced	2	Just outside	Reduced
#3 Miranda	11	≥ 31	≤ 5	High	More	1	Some rooms	No partner
#4 Amelia	15	21–30	6–30	Moderate	Same	2	Just outside	Same
#5 Briana	9	≤ 10	6–30	Low	Reduced	1	Just outside	No partner
#6 Sarah	13	21–30	≤ 5	High	More	3	Nowhere close	No partner
#7 Peta	19	11–20	6–30	Moderate	Reduced	1	Nowhere close	No partner
#8 Rose	13	11–20	6–30	Moderate	Reduced	> 4	Anywhere inside	Same
#9 Sofie	13	≥ 31	≤ 5	High	Reduced	1	Nowhere close	Reduced
#10 Sam	15	11–20	31–60	Low	Same	2	Just outside	No partner
#11 Anna	12	≤ 10	≤ 5	Low	Reduced	1	Just outside	Reduced
#12 Grace	15	≤ 10	6–30	Low	Reduced	2	Some rooms	Stopped
#13 Daniela	13	21–30	≤ 5	High	More	2	Just outside	Same
Average	**13**							

^a^Assessed using the Heaviness of Smoking Index, a two-item self-report measure of dependence based on time to first cigarette and number of cigarettes smoked per day [26]

^b^Some rooms indicates smoking in some but not all rooms in the house; just outside indicates smoking immediately outside the door/window; nowhere close indicates smoking away from the house

### Experiences of smoking

Prior to pregnancy, 10/13 women reported smoking 10 or more cigarettes/day and 6/10 smoked 20–40 cigarettes/day. Smoking to cope with mood, stress, anxiety and boredom was widely acknowledged. Fluctuating patterns of smoking were described, with increased patterns of smoking associated with distressing experiences including intimate partner violence, family conflict and removal of children from care.

*It’s usually the one thing that calms me down. When I used to have panic attacks, like when I did weed, I would have a smoke and then it would all go away…it would just calm me down. And when I have like anxiety attacks now…usually a smoke just like helps me focus on the smoke rather than focusing on my heart beating, or like my palms getting sweaty and freaking me more out. (#12 Grace)*

Other substance use influenced smoking frequency and a link between smoking and cannabis was evident. All cannabis smokers reported mixing tobacco with cannabis, a practice common in Australia to aid consumption and produce a more unique effect, [27] although this was seldomly considered tobacco use by women. Half (6/13) reported waking during the night to smoke tobacco, with incidence varying from infrequently to five or six times overnight.

Overwhelmingly, women disliked smoking tobacco. Most commented on the sensory displeasure including smell, taste, and staining of fingers and teeth. Financial burden and significant impact on health including respiratory-related difficulties, increased nausea, heightened anxiety and broken sleep patterns were noted. Several expressed guilt when smoking around children and pets.

…the effects that it has on my lungs. The coughing, that’s pretty bad. The smell, the taste, the time it actually does take away with having a smoke…smoking in the house, which is something I never used to do…I’ve got a five-year-old daughter and that’s not healthy. So, smoking around her… (#3 Miranda)

I don’t get the pension or anything, it’s only Newstart [government welfare benefits] I’m on…so…we’re left with nothing after practically rent and then your cigarettes. I only get a little bit of food because my cigarettes are more priority than my food…which I don’t want that to be any more. (#1 Nicole)

### Smoking in pregnancy

Most women expressed a desire to stop smoking before birthing. Approximately half (7/13) reported reducing cigarette consumption during the current pregnancy, while others could not or had not tried. A sense that reduction efforts were ‘good enough’ was discerned in those who had reduced. Many (6/9) describing smoking in one or more previous pregnancies.

Well…in the last couple of weeks, it’s [smoking level] maybe three or four a day, so it’s cut completely down and that’s good, it’s not even a full one in one go. I might have half and then put it down and then go back to it later. (#5 Briana)

Women were aware that smoking was harmful for their unborn baby and other children, with this frequently described as motivation to stop. However, most (9/13) acknowledged that they observed these thoughts only occasionally, or when reminded by others. Understanding of specific harms or the extent of harms was less evident.

I thought smoking was actually…smoking was the least of my worries, you know what I mean? (#8 Rose)

Negative self-judgement, shame and stigma was pervasive. Many described doing their best to reduce or spontaneously quit cigarettes and other substances of concern. Several were frustrated that people did not understand that they were doing their best to stop or reduce smoking.

I feel like shit about it. I mean specially now because like, people can see and like, if I'm at work I have one... it's really embarrassing. It's not only that, it's like I don't wanna be smoking when I have the baby and I'm also worried that like…I should have been able to quit by now. (#10 Sam)

At least I cut down on my smokes, and now I've cut down all the alcohol. And I'm reducing my medications, but it's never good enough. (#12 Grace)

Cognitive dissonance and risk denial were apparent as women attempted to rationalize their smoking behaviour.

I absolutely hate it. I have to kind of put it on a scale…yes it calms me down, like throughout everything, but it’s also hurting my baby. So…do I put myself first, or my baby first? Like do I freak out, but my baby’s gonna be healthy or do I calm myself, and it hurts my baby? (#12 Grace)

I don't wanna sound too horrible, but it's kind of like…since I've had other children and I've smoked fulltime and nothing's been wrong, I know it's all chance as well, but... (#5 Briana)

### Smoking cessation

#### Motivations to stop smoking

Most (12/13) expressed an intention to stop smoking before their baby was born. Fetal harm prevention and maternal health were primary motivators, but long-term smoking abstinence to reduce childhood smoke exposure and to be a good role model were frequently cited aspirations.

I know I want to give up for my baby…not just for the baby, for me and for everyone around me. If I can do it, so can they…that’s how I see it. (#1 Nicole)

…the benefits of staying smoke-free after giving birth would be that I'm not providing a bad role model towards my kid…that ‘smoking is okay’…and that every time I go for a smoke that I'm missing time out with my kid. Where I'm being selfish, and I wanna smoke, and say if my kid needs me, I don't wanna leave her unattended, because my partner will be at work. (#12 Grace)

#### Barriers to quitting

Common to all women was the presence of tobacco smokers in their households and social networks who many felt they would receive little support to quit from. Those living in group homes or government housing blocks reported difficulties avoiding others who smoked.

So, I can’t talk to him how I’m feeling about giving up because…he’s not really sympathetic...oh he’s sympathetic, but yeah, it doesn’t help when you’ve got another smoker in your life. (#1 Nicole)

AOD use and heavy nicotine dependence were acknowledged as major reinforcers of tobacco use. The fear of experiencing nicotine withdrawal, including increased stress, anxiety and boredom, deterred quit efforts.

…when I first fell pregnant, I tried to cut back my cigarettes, so smoking less. But unfortunately, I have substance issues or problems as well, so we’ve taken that away and my stress levels have risen. So, my smoking has also increased. (#5 Briana)

[I have a] fear of the whole process of actual quitting because I know it’s not easy, I’ve done it before…what I’m going to be going through, the withdrawing, just feeling lousy and…sleeplessness. I don’t sleep well as it is, and that I know this will increase when I do stop…yeah, you don’t forget. (#3 Miranda)

Stress and anxiety I think, just thinking about it [quitting] makes my anxiety get like, right up. (#4 Amelia)

Hesitancy to use NRT was highlighted. #1 Nicole, #12 Grace and #10 Sam had either stopped or chosen not to use patches because of medication package warning labels discouraging prenatal use. #2 Kate had been told by healthcare providers not to use NRT during pregnancy*.*

To be honest, I do feel like I’ve been smoking more since I found out I was pregnant because, like I took off the patches…I thought they were bad. (#10 Sam)

I know that we can get patches downstairs at the clinic and that, but then…they tell you not to use them as well [during pregnancy] …there's a little bit of mixed information out there. (#2 Kate)

Previous negative experiences also deterred NRT use. Aversion to taste, skin/throat irritations and heartburn were reported, while others felt it did nothing to change their smoking behaviours. The expense of NRT was also prohibitive.

The cravings didn't go down. I still smoked and that's why I didn't really bother to use them…I think I had two patches, so I used them for probably two days. (#5 Briana)

I tried it for a couple of weeks…but the inhalers were just as expensive as buying a packet of cigarettes. (#11 Anna)

#### Strategies used to quit

Most (11/13) had tried to stop smoking at least once, lasting between 2 days and 9 years. Most fell between 2 and 12 months.

The longest I've gone without a cigarette…except when I'm asleep…is like two hours and then I get really cranky and…if I don’t have any, I will go around and ask somebody for a cigarette. (#1 Nicole)

I've tried to stop smoking heaps, you know, cut back and stuff. But yeah, my stress levels go up and I don't know how to cope with it, so, back to the smoke... (#13 Daniela)

NRT was widely used as a cessation support. Long-acting transdermal patches were most common, followed by short-acting gum and inhalers. Feedback on their effectiveness varied.

The inhalers and stuff…and I’ve used the patches but they just…I don’t know, they don’t…and the inhalers they don’t do shit, not for me anyway. (#2 Kate)

I’ve used patches. That was when I successfully quit for four years. (#3 Miranda)

Tobacco counselling, e-cigarettes (vaporised nicotine), telephone support lines, hypnotherapy, exercise, sweet mints and inpatient AOD withdrawal admissions (where hospitals are smoke-free environments) had also assisted quit attempts. Cessation medications (varenicline, bupropion) had been used by 2/12 outside pregnancy. Half (6/13) had attempted to quit ‘cold turkey’ at least once.

### What strategies might help to stop smoking?

When asked what might help to quit smoking, NRT was the most common response (6/13) from women. Extra support, specialized counselling, education, motivational enhancement, distraction, partner support and combined approaches were also suggested. Several women wanted to avoid people or places where smoking was prevalent. Several articulated what would not work, citing telephone support lines, pressure from partners/family and enforced smoking abstinence.

I need some professional to tell me, or like my sister who’s a nurse and who knows her stuff, to tell me. (#12 Grace)

I just need that right push…and the support behind me to give it up. (#1 Nicole)

Opinions were also sought on possible components of a tobacco intervention, specifically financial incentives for smoking abstinence, subsidized/free NRT, telephone-delivered tobacco counselling, and support for partners who smoked.

#### Financial incentives

The idea of receiving money for smoking abstinence was novel and reactions varied. Most (10/13) felt that incentives could provide motivation to stop although others felt that self-motivation should be used. Concerns that people might misrepresent abstinence to receive money and continue smoking were also raised.

I think it’s like a nice reward, because I haven’t given myself any rewards for quitting things. (#12 Grace)

It also is important that you can’t cheat, you know what I mean? Otherwise, I probably would be inclined to. (#8 Rose)

Many (7/13) preferred retail store vouchers over cash rewards primarily because vouchers were more likely to be spent on practical items.

…don’t get me wrong, I’d love the cash in my pocket…but you know, like for what you want to achieve kind of thing that [vouchers] would be more useful and the cash in your pocket is like…you know, if I really need a packet of cigarettes, I can go and buy one. (#13 Daniela)

Few (3/13) felt that weekly amounts over $A100 ($US70) were required to motivate smoking behaviour change. Several (3/13) were unsure and all others (7/13) agreed that $A50 to $A100 was an adequate amount, being roughly equivalent to their weekly cigarette expenditure.

“…well of course…you’re gonna want more but, even like less than fifty dollars is good… I don’t have heaps of money.” (#10 Sam)

#### NRT

All women were receptive to using NRT to assist cessation, particularly if they were to receive it for free and could access all available forms.

The ones that are available on prescription because I have a healthcare card are affordable. But the spray for example isn't covered by the national health so that is unaffordable to me, so if I was given one that would be appreciated. (#7 Peta)

#### Counselling

Many (10/13) agreed that discussing smoking with a professional might be helpful but stressed the counsellor and approach were important. A non-judgmental approach offering encouragement and information about nicotine withdrawal and benefits of quitting were thought to help. Concerns relating to delivery by telephone were identified. Although all had access to a mobile phone, suggestions including having pre-organized call times, using recognizable numbers and using a brief and/or infrequent calling regime were made to counter these.

Yeah, I’m not sure whether that would be a help or a hindrance. (#11 Anna)

…as long as they’re not like people attacking me like my partner, so it’s like a nice thing, they’re trying in a nice way to like, educate me. (#4 Amelia)

#### Partner support

Women with partners who smoked (3/5) thought that NRT supplied to partners would be helpful and welcomed. All acknowledged that they would like partners to join them in counselling, but that their partner may not be willing to participate.

#### Incorporating tobacco treatment into antenatal AOD care.

Nearly all (12/13) women considered tobacco treatment provided as part of their substance use in pregnancy antenatal care acceptable. They cited rapport with clinicians, particularly their non-judgemental and compassionate attitudes, and convenience of attending one setting.

### Clinicians surveys

Participant characteristics of the 28 survey respondents are described in Table 3, survey questions and results are in Table 4.

**Table 3:** Clinicians’ demographic characteristics (n = 28)

Category	Demographic		
Age (*Mean, S)*		47	9
Gender *(%, N)*	Female	89%	25
Role *(%, N)*	Midwife/Nurse	57%	16
	Allied Health Professional	29%	8
	Addiction Specialist	7%	2
	Other	7%	2
Smoking status *(%, N)*	Never	54%	15
	Formerly	43%	12
	Current	4%	1
Smoking Cessation training *(%, N)*	Employment-based education	57%	21
	Self-guided—evidence based resources	32%	12
	Postgraduate education	11%	4
	Free online training (e.g., Quit Victoria)	5%	2
	None	3%	1

Clinicians who responded to the survey were primarily female nursing or allied-health professionals, with all but one having completed some tobacco treatment training. Most were motivated and confident to support their clients to address tobacco smoking behaviours and overwhelmingly, they felt that reducing tobacco use as opposed to tobacco cessation/abstinence-based approaches were appropriate for this group. These could include strategies such as reducing tobacco consumption or switching to non-tobacco containing products such as NRT, medications or vaporized nicotine.

Clinicians agreed that improving the baby’s health, the need to save money and a desire to better their own health were the main motivators for women to stop smoking. The most frequently cited barriers to cessation were stress, smoking by women’s partners and others in close social or familial networks and having to stop more than one substance concurrently. The most effective interventions were considered to be designed specifically for this group of women and include extra behavioural or pharmacological support or a combination of evidence-based strategies (these included support for mental health concerns, relapse prevention, NRT for women and other household smokers, financial incentives).

In open-ended responses, clinicians stated that ongoing maternal nausea and fears about sudden unexpected death in infancy frequently motivated smoking cessation. Stopping cannabis use, admission to smoke-free rehabilitation facilities, clinician-provided cessation advice and carbon monoxide assessments were also thought to encourage cessation.

The lack of access to NRT, particularly misperceptions around maternal use and unaffordability were also cited in open responses. Subsidized NRT in Australia requires a doctor’s prescription for a 12-week supply of patches, gum or lozenges, which was viewed as problematic. Clinicians noted that some healthcare providers lacked confidence to provide smoking treatment and prioritized co-occurring psychosocial issues. Some felt that a reluctance to cease tobacco and AOD use together, and common risk denial strategies were also detrimental to women achieving abstinence.

Several clinicians noted that additional complexities impeding smoking cessation in this group, including that many increase smoking in response to decreasing substance use, need addressing. Provision of all NRT forms, regular support that includes partners/family members and requires little time or travel commitment, as well as education and encouragement were suggested.

### Results synthesis

In triangulating women’s interviews and clinician’s survey results, there were points of concurrence and some areas of contrast. All women described a desire to stop tobacco smoking. This was confirmed by clinicians and supported by their self-reported attempts to stop smoking. Both groups viewed prenatal harm prevention as the major motivation to quit, followed by maternal health and financial improvements. Women were inspired to be good role models, while clinicians thought women’s desires to be addiction-free and prenatal nausea also encouraged smoking behaviour change. Clinicians highlighted their own role in motivating women to address smoking, although this was not acknowledged by women.

Both groups recognized the impact that AOD use has on women’s smoking behaviours, including the frequent co-use of tobacco and cannabis. Increased smoking after reducing AOD use, and a fear of concurrent substance cessation impeded quit efforts. The reliance on tobacco smoking to reduce stress and boredom, and influence of partners/household members who smoke was seen by both groups as significant barriers to be addressed.

Most pregnant women had used NRT, however, negative experiences, financial constraints and conflicting messaging around maternal use had restricted uptake, adherence and effectiveness. Clinicians identified similar issues among their clients, and some were not convinced of NRT’s effectiveness in this group. Despite these doubts, both groups considered unrestricted access to NRT was an important tool in tobacco treatment.

Although they wanted to stop, most women had reduced or were attempting to reduce their smoking and there were indications that several were content with this. Clinicians similarly favoured harm reduction rather than abstinence-based approaches to addressing smoking. Clinicians also encouraged regular, non-judgmental and women-centred care, combined with NRT and education. Women expressed some hesitancy about counselling, particularly around the style and approach, but identified that they needed extra support. Clinicians supported financial incentives as a tobacco treatment strategy. Women were generally positive about being rewarded and the increased motivation that incentives in the vicinity of $A50—$A100 per week could provide. Both groups agreed that supporting partners to support women’s quit attempts was important. Offering tobacco treatment as part of their antenatal AOD care was considered appropriate and convenient.

## Discussion

This study explored tobacco use and treatment for pregnant women with AOD concerns to support the development of an intervention tailored to their needs. Thirteen pregnant women, most with moderate or high tobacco dependence, and 28 antenatal clinicians were recruited from substance use in pregnancy services in NSW, Australia and surveyed about smoking in pregnancy, motivators and barriers to smoking behaviour change and strategies that might assist maternal cessation. The use of mixed methods provided rich descriptions of women’s smoking experiences that were verified and enhanced by a clinical perspective.

Women reported smoking to modify symptoms of mental ill-health and being heavily influenced by smoking in their living and social environments, characteristics previously reported in high-risk groups of smokers including those vulnerable to mental illness and AOD use [28]. More nuanced were concerns about stopping multiple substances concurrently, shame, stigma and low self-efficacy, overnight smoking (an indicator of heavy dependence), [29] and fear of withdrawal symptoms. Women reported being motivated to stop smoking and most had tried previously to do so without success. Both motivation and prior quit attempts are predictive of future quit attempts, but lower nicotine dependence predicts cessation success [30]. Treatments suitable for highly nicotine dependent pregnant women that increase capability [31], utilize multiple approaches [11] and provide extra support, education and motivation may be required to achieve long-term behaviour change in women with maternal AOD concerns.

Antenatal clinicians almost unanimously favoured a harm reduction approach to tobacco treatment for AOD clients and many women demonstrated this approach by reducing or attempting to reduce cigarette smoking. Tobacco harm reduction cannot produce the benefits that abstinence provides [32] but reducing cigarette consumption may reduce financial harm and has been associated with later quitting [33]. However, in Australia, guidelines recommend encouraging cessation rather than reduction [49]. Clinician preference for harm reduction may be related to accumulated experience with women who are unable or resistant to quitting and their desire to assist women to reduce at least some negative outcomes. Women who are satisfied with reduced smoking may not go on to attempt cessation. Further investigation is warranted as both potentially limit opportunities to achieve abstinence during pregnancy. Several women had tried vaporized nicotine and varenicline prior to pregnancy. Accumulating evidence suggests these have potential to be effective aids to smoking cessation in pregnancy [17, 34] and are also worthy of further exploration in this group.

This group of clinicians were willing to discuss simultaneous cessation of tobacco and other substances. Previous evidence has found that that healthcare providers have prioritised cessation of AODs over tobacco [15] and in some cases, have avoided recommending tobacco treatment early in other AOD treatment for fear that stopping tobacco use may compromise treatment success [13, 35]. Although these were not specific to pregnancy-related treatment. The desire to reduce prenatal harms and the teachable moment pregnancy provides [36], may be motivation to address both substances at once and clinicians should be encouraged to do so, notwithstanding that behaviour change in those treated for substance use disorder is often slow, and characterised by relapse [37]. Most surveyed clinicians had training and confidence to deliver smoking treatment but reported inconsistencies among other healthcare providers. Lack of skills and confidence to support smoking cessation is common and undermines women’s success [38]. Conflicting priorities, such as the need to focus on unstable accommodation, family and domestic violence or child protection issues, may also hinder the clinical management of tobacco use [39]. Enhancing clinician capability and their ability to provide holistic care should be addressed to improve treatment outcomes for women with AOD concerns.

Provision of NRT was strongly encouraged by both groups despite hesitancy around its effectiveness, risk and accessibility. Negative experiences with NRT are known to hinder its use in pregnancy [40]. This could be mitigated by clear messaging, education on correct use in pregnancy and adequate dose [41] and subsidized or free access to all NRT [42]. Women consider professional advice when deciding whether to use NRT [40], so risk–benefit and harm reduction discussions with clinicians are important. Combination therapy (using long and short-acting forms together) and encouraging women to self-titrate doses to alleviate cravings will enhance effectiveness [43, 44] especially in those with higher nicotine dependence.

Support for counselling and financial incentives was mostly positive. Resistance to psychosocial interventions is not uncommon in AOD populations and can be related to shame and stigma [45]. Education and motivational enhancement were considered important by women and are integral to counselling-based therapy, so labelling them as such may help. Incentives increase the long-term rates of cessation in pregnant smokers [46] although their utility as a smoking cessation strategy in opiate-dependent pregnant women showed limited success [19]. Their effectiveness in combination with other supports such as NRT and counselling has not been trialled in this population and could improve overall outcomes. Concerns about the potential for deception would be minimized by verification with breath carbon monoxide.

### Implications for practice, policy and research

Tobacco smoking prevalence remains elevated in high-priority groups such as pregnant women with substance use concerns, where the burden of harm is disproportionately higher than in general populations [47]. Treatments designed to meet the unique needs of such groups are essential to reduce the overall burden of tobacco-related harm. The information captured by this study verifies the necessary components of a targeted tobacco treatment incorporating subsidized and individualized NRT (including education on correct use), financial incentives for verified abstinence, psychoeducation for smoking cessation and additional support for partners. The next steps would include pilot testing of the treatment to ensure its feasibility and acceptability in healthcare settings.

### Strengths and limitations

The combination of consumer and healthcare provider perspectives and mixed-methods research provides a detailed and nuanced understanding of smoking characteristics, cessation motivations and barriers and treatment options for pregnant women with AOD concerns. The sample of women demonstrated diversity across most demographic and tobacco related characteristics including tobacco dependence and substances used/prescribed, education, parity and gestational age. It is broadly reflective of clients seen at participating clinics, although this sample is slightly older (33 years vs. 29 years) and has a smaller representation of those who use methamphetamines when compared to previously published attendance of one included site [10, 48]. The characteristics, however, are limited by a lack of cultural data that was overlooked during data collection. They are also not reflective of all pregnant women and the inferences drawn here may not be generalizable to these or other specific subsets or disadvantaged groups.

The structured nature of the clinician survey limited interpretation of some results. The possibility of respondent bias should be considered as the majority of those surveyed were advocates for maternal smoking treatment, who had received training and were confident in their treatment abilities. The perspective of those less skilled and motivated was lacking, potentially impacting broader generalizability of results. Survey responses were also limited by eligibility restrictions to those working in current clinical antenatal roles and precluded some others, such as service managers. Sample sizes in both groups are small but should be considered relative to their modest population size when drawing conclusions or generalizing results to other population groups.

## Conclusion

This study combines consumer and healthcare provider insights to explore the complex relationship between tobacco smoking and other substance use, enhancing our understanding of smoking treatment for pregnant women with substance use concerns. The combined treatment perspective provides a scaffold for the development of a targeted tobacco treatment incorporating NRT, financial incentives, education and counselling as well as partner support. As in other AOD interventions, a non-judgmental approach and a harm reduction framework are necessary foundations. Knowledge and skill development of clinicians to encourage eventual tobacco abstinence and simultaneous reduction or cessation of AOD would also facilitate successful treatment outcomes.

### Supplementary Information

**Additional file 1.** COREQ checklist.

**Additional file 2.** GRAMMS checklist.

**Additional file 3.** Women's interview schedule.

**Additional file 4.** Women's interview codebook.

## Data Availability

The data that informs the findings of this study is available from the corresponding author (MAJ) upon reasonable request.

## Appendix

**Table 4:** Clinician survey questions and responses (n=28)

1. How much do you agree or disagree with the following statements?	Agree %	Disagree %	Unsure %
•I feel confident in my knowledge about the harms of smoking to the fetus to discuss them effectively	93	3	3
•I feel confident in my knowledge of the harms of secondhand smoke exposure on infants and children to discuss them effectively	93	3	3
•A harm-reduction approach should be used when addressing smoking (i.e. reduce tobacco consumption, switch to non-tobacco containing products e.g. NRT, electronic cigarettes)	93	0	7
•Brief smoking cessation advice (e.g. 5A's, motivational interviewing, education) is effective in addressing tobacco use	73	20	7
•An AOD-based antenatal service is an effective place to implement a smoking cessation intervention	70	13	17
•These clients generally want to stop smoking but don't have the skills/resources to do so	63	37	0
•It is not my role to provide smoking cessation treatment to these clients	20	80	0
•An abstinence approach should be used when addressing smoking (i.e. quit all nicotine/tobacco)	13	87	0
•It is too difficult for these clients to stop smoking and other substance use together, so I wouldn't suggest it	7	87	7

Women’s motivators for smoking cessation	
2. How often do you see or hear the following motivators or reasons to stop smoking?	Sometimes / Often %
•The desire to improve baby's health outcomes	97
•The need for more disposable income or to save money	72
•The desire to be free of addiction to all substances	69
•The wish to improve physical and/or mental health	66
•Support and encouragement provided by healthcare providers	66
•Pressure from partner, family members or friends to stop	55
•The desire to remove cigarette-smoke odors from house, car etc	45
•The dislike of tobacco smoking	34
•The desire to avoid the stigma-laden reactions of others to smoking while pregnant	31
•The wish to improve hygiene e.g. clean breath, clean fingers, white teeth	28

Women’s barriers to smoking cessation	
3. How often do you see or hear the following barriers to smoking cessation?	Sometimes / Often %
•Tobacco smoking is a way of coping with stress	100
•Having a partner who smokes	97
•The belief that it is too difficult to stop smoking and other substances at the one time	97
•The enjoyment of tobacco smoking	93
•The acceptability of smoking within client's social circles—'…everyone around me smokes'	90
•Tobacco smoking helps to relieve boredom	76
•Little understanding of the health consequences of cigarette toxins on baby's health outcomes	76
•Concerns about withdrawal symptoms e.g. irritability, increase in anxiety/depression symptoms	72
•The belief that tobacco is not illegal so is not as important to stop as other substances	66
•The prohibitive cost of pharmacotherapy treatments e.g. NRT	66
•Little or no cessation advice or support given by health service providers	48
•Concerns about the likelihood of weight gain	41
•The belief that smoking may lead to reduced baby size and an easier delivery	38

Effective smoking interventions				
4. How effective do you believe it is to include the following into a smoking treatment tailored to this group?	Not %	Some what %	Very %	Unsure %
•Women-centered care (i.e. treatment focused on a woman's unique needs)	0	21	68	11
•Support (behavioural or pharmacological) for substance use	0	32	64	4
•A combination of the above strategies	0	25	64	11
•Support (behavioural or pharmacological) for mental health issues	0	36	57	7
•Postpartum smoking relapse prevention	0	36	57	7
•Supply of NRT for partners or other household members who smoke	0	43	50	7
•NRT	0	54	43	4
•Financial incentives to stop smoking	4	32	43	21
•Facilitation of social support (e.g. quit buddy or Quitline referral)	14	46	29	11

## Abbreviations

NRT: Nicotine replacement therapy
AOD: Alcohol and other drugs
NSW: New South Wales
