# Supplementary material for: Using mixed methods to establish tobacco treatment acceptability from the perspective of clients and clinicians of antenatal substance use services
Source: Addict Sci Clin Pract. 2022 Oct 4;17:56. doi: 10.1186/s13722-022-00337-y (PMC9531520; doi:10.1186/s13722-022-00337-y)
Supplement: Supplementary file 1 — Additional file 1. COREQ checklist. [file 13722_2022_337_MOESM1_ESM.docx]

# Developing a smoking intervention for pregnant women who smoke tobacco and use other substances: A qualitative enquiry regarding barriers and facilitators to quitting

***Opening blurb:***

Thank you for agreeing to talk with me today. I want you to tell me about your thoughts and opinions around tobacco smoking. I want to understand what influences your smoking, your feelings about stopping and what you think may help you to stop in the future.

There are no right or wrong answers to any of the questions I ask. The information you provide will help us create smoking cessation programs to be used in the future as part of the antenatal care offered here at the clinic.

As was discussed in the information sheet, our talk will be audio-recorded. All the information that you provide will be treated confidentially, unless you reveal information to me of a nature that I am bound by law to report like issues concerning child protection and domestic and family violence.

Please let me know if you would like to stop the interview at any stage. If you want any of your comments deleted from the recording, tell me when we have finished the session, or later on if necessary. The Information Statement that you received has contact details of the researchers involved with this study should you wish to contact us. It also has information about the ethics committee who approved the study should you have any problems relating to the research. Do you have any questions or is there anything that you don’t understand?

After we have finished, I will give you a short questionnaire to complete. It will ask you a few questions to give us some information about your background and smoking history. The answers you give will be combined with the information of others and will not identify you in any way.

***Interview questions***

# The following questions and suggested prompts (provided below each question) are to be used as a guide to explore the following themes regarding tobacco smoking:

1. **Experiences of smoking and smoking cessation**
2. **Attitudes to smoking and smoking cessation while pregnant**
3. **Facilitators and barriers to smoking cessation**
4. **Strategies to enhance smoking cessation**

- **Can you tell me about your smoking history and your smoking patterns now?**
  - *When did you start smoking?*
  - *How much do you smoke?*
  - *When, or with who, do you smoke the most?*

# What do you like most about smoking and what do you like the least?

- - *Positives of smoking?*
  - *Negatives of smoking?*

# How do you feel about smoking now that you are pregnant?

- - *Why do you think that people make such a fuss about pregnant mums smoking?*
  - *Have you smoked in any other pregnancies? If so, how much?*
  - *How often do you think about the effects of smoking on you and your baby?*

# What are your thoughts about stopping smoking while you are pregnant?

- - *What would be good about stopping smoking now?*
  - *What would be the benefits of staying smoke free after your baby is born?*
  - *How do you feel about stopping smoking rather than just reducing it?*
  - *What are some of the things that stop you from giving up smoking now?*
  - *How does your use of other substances influence your smoking?*

# What is your experience of trying to stop smoking?

- - *How many times have you tried in the past?*
  - *How long did you last?*
  - *What did you use to help you stop (cold turkey, behavioural counselling, NRT, e-cigarettes, quitting medications e.g. Champix, Zyban, hypnotherapy, financial rewards, Quit line, others)?*
  - *What was most helpful for you in helping to stop?*
  - *What was least helpful for you in helping to stop?*

# Are there other people in your household who also smoke? If not, is there a person close to you, who you see regularly, that also smokes?

- - *How is your smoking influenced by others in your home, or close to you, who also smoke?*
  - *How have they helped you to stop smoking in the past?*
  - *How have they made your attempts to stop harder?*

# What strategies for quitting smoking do you think would work for you?

- **What do you think about receiving regular support calls by an experienced smoking counsellor to help you to reduce or stop smoking?**
  - *How would this be helpful for you now?*
  - *What problems could you think of that might make it difficult to take these support calls?*
  - *Would you like to have these types of sessions with your partner present as well?*

# How would you feel about receiving a free supply of NRT (like patches, gum, inhalers etc.) to help you reduce or stop smoking during your pregnancy?

- - *What types of NRT have you used in the past?*
  - *Which ones did you not like? Why?*
  - *If you haven’t ever used them, what are the reasons?*
  - *How helpful do you think NRT would be for your partner (or other household smokers)?*

# How do you feel about being rewarded with gift vouchers to stop smoking? For example, every week that you don’t smoke, you receive a voucher?

- - *How much would financial rewards help you to* ***stop*** *smoking?*
  - *How much would they help you to* ***reduce*** *your smoking?*
  - *Would you prefer supermarket and discount store vouchers or cash?*

# Do you think is appropriate that the health service pay people to quit smoking?

- **How much of a reward would make you think about giving up smoking?**
  - *Values less than $50 per week, between $50 and $100, or over $100 per week?*
  - *Why this amount?*
  - *What would this amount of money do for you or your family each week?*

# How do you feel about being offered help to quit smoking while you attend the Tuesday afternoon high risk antenatal clinic?

- - *Why would this be a good place to offer support for you to quit while pregnant?*
  - *Do you feel comfortable talking with the doctors and nurses at the clinic about smoking?*
  - *Is there other places or agencies that you think might do a better job of supporting you to quit or reduce smoking while you are pregnant?*

# Is there anything else you’d like to say about smoking or what may help you to stop?

**Demographic Questions**

1. **What is your year of birth?**
2. **How far did you get in your education? *(tick one only)***
   - Primary school and up to year 9
   - Up to and including Year 10
   - Up to and including Year 12
   - TAFE certificate or diploma
   - Bachelor’s degree/Post-graduate degree
3. **Do you have a partner?**
   - Yes
   - No
4. **What is your main source of income?**
   - Full-time work
   - Part-time/casual work
   - Temporary benefit (i.e. NewStart)
   - Parenting Payment
   - Pension (i.e. disability or veterans)
   - No income/dependent on others
5. **How many children do you have (that you gave birth to)?**
   - 0 ☐ 1 ☐ 2 ☐ 3 ☐ 4 ☐ 5 + (*please specify number) _________*

# How many children are usually living in your household?

- - 0 ☐ 1 ☐ 2 ☐ 3 ☐ 4 ☐ 5 + (*please specify number) _________*

# How far are you into your pregnancy (in weeks)? /40

1. **Which of the following best describes your smoking status?**
   - I stopped smoking after I found out I was pregnant, and I am not smoking now
   - I smoke some now, but I cut down on the number of cigarettes I smoke since I found out I was pregnant
   - I smoke regularly now, about the same as before I found out I was pregnant
   - I smoke more now than I did before I was pregnant
2. **How old were you when you first had a cigarette?**
3. **How often do you smoke?**
   - Every day
   - Most days (four or more days a week but not every day)
   - Occasionally (3 times or less a week)
4. **How many cigarettes do you smoke on a day when you do smoke?**
   - 10 or less

☐ 11-20

☐ 21-30

- - 31 or more

1. **How soon do you have your first smoke after waking in the morning when you do smoke?**
   - Within 5 minutes
   - 6-30 minutes
   - 31-60 minutes
   - Over 60 minutes
2. **Which substance (other than tobacco) do you use most often? *(tick only one)***
   - Alcohol ☐ Amphetamine/methamphetamine
   - Benzodiazepines ☐ Buprenorphine
   - Cannabis ☐ Methadone
   - Synthetic Cannabis ☐ Heroin
   - Other opioids (e.g. OxyContin, OxyNorm, Endone, MS Contin, Kapanol, MS Mono, fentanyl patches, codeine)
   - Other
   - None
3. **Which substances (other than tobacco) do you use less regularly? *(tick as many as apply)***
   - Alcohol ☐ Benzodiazepines
   - Amphetamine/methamphetamine ☐ Heroin
   - Cannabis ☐ Methadone
   - Synthetic Cannabis ☐ Buprenorphine
   - Other opioids (e.g. OxyContin, OxyNorm, Endone, MS Contin, Kapanol, MS Mono, fentanyl patches, codeine)
   - Other
   - None
4. **How many smokers usually live in your household? (DON’T include yourself)**
   - None
   - One

☐ 2-3

- - More than 3

1. **How does your household manage places where smoking is allowed?**
   - People can smoke anywhere
   - Smoking is only allowed in some rooms inside
   - Smoking is allowed on the veranda or just outside the door/window, but not inside
   - No smoking is allowed inside and no smoking just outside the house
2. **Which of the following best describes the smoking status of your current partner?**
   - He/she have never smoked or have smoked fewer than 100 cigarettes in their lifetime
   - He/she stopped smoking before I found out I was pregnant, and are not smoking now
   - He/she stopped smoking after I found out I was pregnant, and are not smoking now
   - He/she smoke some now, but have cut down on the number of cigarettes they smoke since I found out I was pregnant
   - He/she smoke regularly now, about the same as before I found out I was pregnant
   - He/she smoke more now than I did before I found out I was pregnant

***Thank you for sharing your experiences and your time and for taking part in our research.***
