# Supplementary material for: Using mixed methods to establish tobacco treatment acceptability from the perspective of clients and clinicians of antenatal substance use services
Source: Addict Sci Clin Pract. 2022 Oct 4;17:56. doi: 10.1186/s13722-022-00337-y (PMC9531520; doi:10.1186/s13722-022-00337-y)
Supplement: Supplementary file 2 — Additional file 2. GRAMMS checklist. [file 13722_2022_337_MOESM2_ESM.docx]

**Good Reporting of a Mixed Methods Study (GRAMMS) checklist**

| **Guideline** | **Section: page** |
| --- | --- |
| Describe the justification for using a mixed methods approach to the research question | Methods – Design & Settings p5 |
| Describe the design in terms of the purpose, priority and sequence of methods | Methods – Design & Settings p5 |
| Describe each method in terms of sampling, data collection and analysis | Described separately Design & Settings p5-7 |
| Describe where integration has occurred, how it has occurred and who has participated in it | Methods – Design & Settings p5 |
| Describe any limitation of one method associated with the presenting of the other method | Discussion – Strengths & limitations p15 |
| Describe any insights gained from mixing or integrating methods | Results synthesis p12-13  Strengths & limitations p15 |
