# Supplementary material for: Using mixed methods to establish tobacco treatment acceptability from the perspective of clients and clinicians of antenatal substance use services
Source: Addict Sci Clin Pract. 2022 Oct 4;17:56. doi: 10.1186/s13722-022-00337-y (PMC9531520; doi:10.1186/s13722-022-00337-y)
Supplement: Supplementary file 4 — Additional file 4. Women's interview codebook. [file 13722_2022_337_MOESM4_ESM.docx]

Womens Interviews_Codebook_MJ

## Nodes

| **Name** | **Description** | **Files** | **Refs** |
| --- | --- | --- | --- |
| 1. Smoking experiences |  | 0 | 0 |
| 1. Smoking initiation | Captures age and context around starting smoking. | 11 | 13 |
| 2. Fluctuations in smoking over time | Recognises that smoking levels are often not consistent and change over time. | 0 | 0 |
| Current smoking | Any references to current smoking patterns | 11 | 21 |
| Pre-pregnancy | Covers all historical references to smoking and how it has changed over time. | 11 | 34 |
| 3. Smoking context & circumstances | The where, when and how of smoking in women’s lives - Including pregnancy? | 11 | 74 |
| 2. Smoking attitudes | To differentiate between experiences. Covers likes, dislikes and ambivalence around smoking. | 0 | 0 |
| Smoking ambivalence | Added to cover situations where women are unsure why they are smoking or what they like about it. | 6 | 14 |
| Smoking dislikes | Covers all aspects of smoking that women mention they don’t like. | 11 | 38 |
| Smoking purpose, need, function | Code began as ‘Smoking likes’ but women did not ‘like’ smoking. This now covers references to the things that maintain smoking and why. | 11 | 22 |
| 3. Smoking and pregnancy |  | 0 | 0 |
| Effects on baby | Recognition of the effects that smoking has on unborn baby and children | 11 | 29 |
| Effects on health | Recognition of the effects that smoking has on women themselves | 7 | 13 |
| Social & Moral Judgements | Created to encompass discussions around stigma, the negative views of other people in relation to smoking, and internalised judgements of shame and guilt. | 11 | 41 |
| 4. Smoking cessation |  | 0 | 0 |
| i. Quitting context & circumstances | When and why of quitting - what were the factors/events that influenced previous quit attempts | 11 | 37 |
| ii. Aspirations & motivations | Comments that capture the feelings around wanting to or believing they are capable of stopping cigarette smoking. I have excluded self efficacy and now covers womens aspirations and motivations for not smoking. Self efficacy is now seperate. | 11 | 73 |
| iii. Self-efficacy | Created a node on its own to cover both positive and negative self efficacy, or how much of a belief a women has in her ability to stop smoking. | 11 | 30 |
| iv. Smoking reinforcers & barriers to quitting | The things, events or people that make it difficult to stop smoking | 11 | 76 |
| v. Strategies used to stop or reduce | To identify what they have used or planned to use to support a quit attempt or reduction previously, including small helpful strategies or evidence based intervientions | 11 | 67 |
| vi. Support of Others | To capture information about anticipated or experienced support of others in trying to quit smoking. | 8 | 19 |
| vii. What would help to stop smoking | What women belive would help them to stop smoking. There is overlap with 4(v). This question was mandated by Hunter New England Health Human Research Ethics Committee (HNE HREC) | 8 | 13 |
| 5. Intervention strategies |  | 0 | 0 |
| *Counselling* |  | 0 | 0 |
| Accessability | Covering ability to take calls - phone, data etc. | 8 | 12 |
| Agree or disagree | Thoughts around the idea of counselling as a quit strategy | 10 | 19 |
| *Financial rewards* |  | 1 | 1 |
| Agree or disagree | Thoughts around the idea of contingency management as a quit strategy | 10 | 24 |
| Amount | The amounts of money that might motivate behaviour change. | 10 | 19 |
| Cash v vouchers | Thoughts around the idea of receiving cash or vouchers as incentives. | 7 | 7 |
| Ethics | Concerns the ethical dilemma about whether a health service should ‘pay’ it’s patients to stop smoking. This question was mandated by HNE HREC. | 10 | 11 |
| *NRT* | Some crossover here. NRT is often discussed as part of a cessation attempt and not part of the potential intervention. Discuss with PB. Added pharmacotherapy to cover Champix etc. | 0 | 0 |
| Agreeable to use | Would the NRT be something that women would use or find helpful to stop smoking? | 9 | 10 |
| Misinformation | There are conflicting views around the use, amounts to use and efficacy of NRT in pregnancy. This has resulted in people being misinformed and this captures those thoughts and comments. | 3 | 4 |
| NRT for partner | Would NRT be helpful for partners or other household members to stop/reduce smoking or support women to stop/reduce smoking? | 3 | 5 |
| Past experience with NRT | Comments on previous expereinces using NRT – overlaps with 4(v). | 10 | 28 |
| *Suitable services* | Is the antenatal service a suitable place to offer this smoking intervention? | 10 | 14 |
| 6. Miscellaneous |  | 5 | 7 |
